# Standardization of a Model of Vertebral Metastasis of Breast Cancer in CD1/Nu/Nu Mice

**DOI:** 10.7759/cureus.77291

**Published:** 2025-01-11

**Authors:** Gervith Reyes Soto, Vladimir Miranda-Galván, Norma Uribe-Uribe, Juan Manuel Escobar-Valderrama, Jorge Alanis-Mendizabal, Luis A Medina-Velázquez, Alejandro Garcia, Gonzalo Torres Villalobos, Fabian Díaz-Martínez, Paola Montiel de la Rosa, Carlos Bravo-Reyna, Alejandra Guadalupe Cervantes Zentella, Geovanny Jose Vanegas Cerna, Vladimir Nikolenko, Tshiunza Cherubin, Andreina Rosario Rosario, Carlos Castillo-Rangel, Mario Antonio Furcal Aybar, Laith Wisam Alsaed, Manuel De Jesus Encarnacion Ramirez

**Affiliations:** 1 Neurosurgical Oncology, Mexico National Cancer Institute, Tlalpan, MEX; 2 Experimental Surgery, Instituto Nacional De Ciencias Médicas Y Nutrición Salvador Zubirán, Mexico City, MEX; 3 Pathology, Instituto Nacional De Ciencias Médicas Y Nutrición Salvador Zubirán, Mexico City, MEX; 4 Basic Research, Instituto Nacional De Cancerología (INCan), Mexico City, MEX; 5 Biochemistry, Instituto Nacional De Ciencias Médicas Y Nutrición Salvador Zubirán, Mexico City, MEX; 6 Physiology and Cell Development, Instituto Nacional De Perinatología, Mexico City, MEX; 7 Neurosurgery, Hospital Bautista, Managua, NIC; 8 Human Anatomy and Histology, N.V. Sklifosovskiy Institute of Clinical Medicine, I.M. Sechenov First Moscow State Medical University (Sechenov University), Moscow, RUS; 9 Neurosurgery, Clinique Ngaliema, Kinshasa, COD; 10 College of Medicine, Autonomous University of Santo Domingo (UASD), Santo Domingo, DOM; 11 Neurosurgery, Hospital Regional 1° De Octubre, Instituto De Seguridad Y Servicios Sociales de Los Trabajadores Del Estado, Mexico City, MEX; 12 Oncological Surgery, Instituto Nacional Del Cáncer Rosa Emilia Sánchez Pérez De Tavares (INCART), Santo Domingo, DOM; 13 Neurosurgery, Russian People's Friendship University, Moscow, RUS; 14 Human Anatomy and Histology, N.V. Sklifosovskiy Institute of Clinical Medicine, Moscow, RUS

**Keywords:** 4t1, breast cancer, hematogenous metastasis, pain, pet-ct, spinal cord compression

## Abstract

Introduction: Breast cancer is the leading cause of cancer-related death in Mexico, with high mortality associated with spinal bone metastasis. We propose to standardize a murine model of bone metastasis to study and understand the tumor microenvironment.

Materials and methods: An experimental, prospective, longitudinal study was conducted using 18 CD1/Nu/Nu 30g nude mice. Two cell lines, MCF-7 and 4T1, were inoculated, clinical follow-up was performed, and biopsy samples were obtained for histopathological evaluation.

Results: Histopathological evaluation of models inoculated with the MCF-7 cell line showed no tumor development, while inoculation with the 4T1 cell line resulted in tumor development, as evidenced by PET-CT and histopathology, using 5,000 and 1,000 cells, respectively.

Conclusions: The use of this model is proposed for studying the clinical, molecular, and prognostic aspects of breast cancer progression by inoculating 1,000 cells of the 4T1 cell line.

## Introduction

Breast cancer is the most frequent neoplasm in women worldwide with a frequency of 1.67 million new cases annually [1]. It is estimated that around 90% of breast cancer deaths are caused by metastasis, with bone tissue being the system with the highest incidence (65-75%) [2]. The percentage of patients with metastasis to the spine is 15% to 20%. The associated symptoms include pain due to spinal cord compression, fractures of vertebral bodies, and/or neurological sequelae due to cellular proliferation [3].

The bone system expresses several growth factors, such as insulin-like growth factor type 1 (IGF-1), fibroblast growth factor (FGF), platelet-derived growth factor (PDGF), transforming growth factor beta (TGF-β), vascular endothelial growth factor (VEGF), hypoxia-inducible factor (HIF), and some cytokines, like interleukin 11 (IL-11), as well as chemokines, including monocyte chemotactic protein 1 (MCP-1), which are associated with the development of a microenvironment that supports tumor cell proliferation [2,4,5]. In addition, bone metastases are facilitated by the high vascularization in long bones such as the pelvis, ribs, and vertebrae. The lumbar vertebrae are the most affected, followed by the thoracic, cervical, and finally the sacral vertebrae [6-9].

Bone, as mentioned, is the most prevalent site of metastasis in breast cancer. Despite the high rate of organ metastasis, patients with metastases to bone have a better prognosis than patients with metastases to visceral organs [10,11]. Currently, there are no laboratory or imaging tests that provide an early diagnosis of the presence of bone metastasis, as many patients are asymptomatic and it takes a long time for symptoms to appear. Molecular tests are now required to provide an early and accurate diagnosis for the detection of bone metastases [12]. Previously, breast cancer metastasis models have been developed using the 4T1 cell line; however, there is no model specific to spine metastasis [13].

We developed a mouse model of breast cancer metastasis in the spine with the aim of studying the early detection of this metastasis through the identification of cytokines expressed due to changes in the microenvironment caused by the presence of tumor cells in bone tissue, as well as testing various drugs that may limit the damage caused by spinal cord compression resulting from breast cancer metastasis in the spine.

## Materials and methods

Study design and animal model

A prospective, longitudinal experimental study was performed to standardize a vertebral metastasis model of breast cancer using female CD1/nu/nu nude mice. The study adhered to the ethical guidelines set forth by the NOM 062-ZOO-1999, and the biotherium provided the necessary permissions. Eighteen clinically healthy female nude mice weighing approximately 30 g were used in the study.

Cell lines and preparation

Two breast cancer cell lines were used: the human MCF-7 cell line and the murine 4T1 cell line. The cells were cultured in a medium containing 10% fetal bovine serum, 1% penicillin-streptomycin-neomycin, and 10 µg/mL bovine insulin, maintained at 95% humidity, 5% CO_2_, and 37°C. Prior to inoculation, the cells were washed twice with PBS to remove serum residues and then maintained in the serum-free minimal essential medium for 48 hours. For the inoculation, 15 µmol/L of β-estradiol dissolved in dimethylsulfoxide was used to induce tumor development in the vertebral bodies.

Surgical procedure

The surgical procedures were performed under aseptic conditions using a rodent anesthesia machine (Kent Scientific Somno Suite). Isoflurane was administered at an induction concentration of 5% and maintained at 2-3%. Meloxicam (5 mg/kg) was administered subcutaneously as an analgesic. The mice were placed in the dorsal decubitus position, and antisepsis of the abdominal region was performed with chlorhexidine. A midline longitudinal incision was made, and dissection was carried out by planes to access the abdominal cavity. Using a surgical microscope (Carl Ziess, OPMI-1), the renal vessels and abdominal aorta were identified. The psoas muscle on the left side at the level of L5 was dissected to access the lumbar vertebral body without stimulating the sacral plexus nerves.

**Figure 1:**
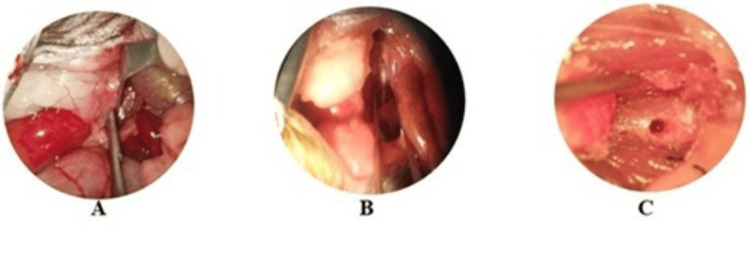
Surgical approach: A) evisceration and identification of the psoas and vascular structures; B) dissection of the psoas muscle and localization of the lumbar vertebral body; C) perforated lumbar vertebral body, exposed for inoculation of cancer cells.

To perform the surgical procedure, the animals were connected to a rodent anesthesia machine (Kent Scientific Somno Suite) using Isoflurane as an anesthetic at an induction concentration of 5% and with a maintenance concentration of 2-3%, as analgesic was administered subcutaneously with a 25G syringe Meloxicam (5 mg/kg) prior to the procedure. The animals were placed in the dorsal decubitus position, and antisepsis of the abdominal region was performed using chlorhexidine. A longitudinal incision was then made along the midline, and dissection was carried out by planes to access the abdominal cavity. With the help of retractors, the viscera were mobilized, and the surgical microscope (Carl Zeiss, OPMI-1) was used. The renal vessels and abdominal aorta were identified, and once located, the psoas muscle on the left side was dissected at the level of L5 to access the vertebral body, taking care not to stimulate the nerves of the sacral plexus running within the psoas. Once the vertebral body was identified, subperiosteal dissection was performed, followed by perforation of the vertebral body to reach the bone marrow, the site of tumor cell inoculation (Figure 1). Finally, the vertebra was covered with bone wax, and closure was performed in layers using 6-0 absorbable sutures. The mouse was then placed in the left lateral decubitus position on a thermal bed for surgical and anesthetic recovery [14].

Cell inoculation

After exposing the vertebral body, subperiosteal dissection was performed, and the vertebral body was perforated to reach the bone marrow. Tumor cells were inoculated at this site: initially, 5,000 cells of the MCF-7 line were used, followed by 4T1 cells in subsequent phases. The vertebra was then covered with bone wax, and the incision was closed in layers using 6-0 absorbable sutures. Post-surgery, the mice were placed in the left lateral decubitus position on a thermal bed for recovery.

Clinical follow-up and endpoints

The mice were monitored for clinical signs weekly, assessing behavior and locomotion parameters such as activity, posture, piloerection, self-mutilation, and intake of food and water. The humane endpoints included signs of severe pain, immobility, and significant weight loss. Upon reaching these endpoints, the mice were sacrificed, and tissue samples from the lumbar region were collected for histopathological evaluation.

Histopathological and imaging analysis

Histopathological evaluation was performed on the collected tissues using H&E staining. For imaging, PET-CT scans using 18-FDG as a contrast agent were employed to detect tumor development and metastasis. The first phase involved the inoculation of MCF-7 cells, which did not result in tumor formation. In the second phase, the 4T1 cell line was used, leading to successful tumor formation and metastasis to the lumbar vertebrae.

## Results

Experimental phases and results

In the first phase with MCF-7 cells, eight surgical procedures were performed. Four mice died (two during surgery and two post-surgery), and the remaining mice showed no tumor development. In the second phase, 4T1 cells were used in eight mice, with two deaths occurring (one during surgery and one post-surgery). The remaining mice exhibited neurological signs such as claudication and isolation by the third week. PET-CT and histopathological analyses confirmed tumor development and metastasis in these mice.

Reproducibility and model validation

After successful tumor induction with 1,000 4T1 cells, two additional surgeries were performed, confirming the reproducibility of the model. The mice displayed clinical and macroscopic changes, and histopathological analysis validated the presence of tumor cells in the spinal column.

In the first phase, eight surgical procedures were performed where 5,000 tumor cells (MCF7) suspended in 5 mL of culture medium were inoculated. There were four deaths; two trans-surgical and two post-surgical (one day after the procedure).

**Figure 2:**
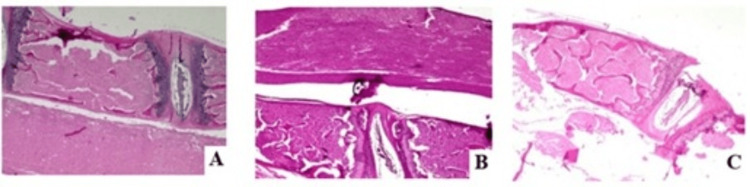
Histopathological sections with H&E staining of the spinal column of mice inoculated with breast cancer cells from the human MCF-7 cell line, showing cells with a necrotic appearance in the bone marrow, without identifying tumor-origin cells. A) Mouse 1; B) Mouse 2; C) Mouse 3.

At the end of the study, the animals were sacrificed, and biopsies of the lumbar region were obtained for preservation and processing for histopathological evaluation using H&E staining. The obtained images showed the bone and marrow tissue without significant changes, except for necrotic areas, with no presence of tumor cells (Figure 2).

**Figure 3:**
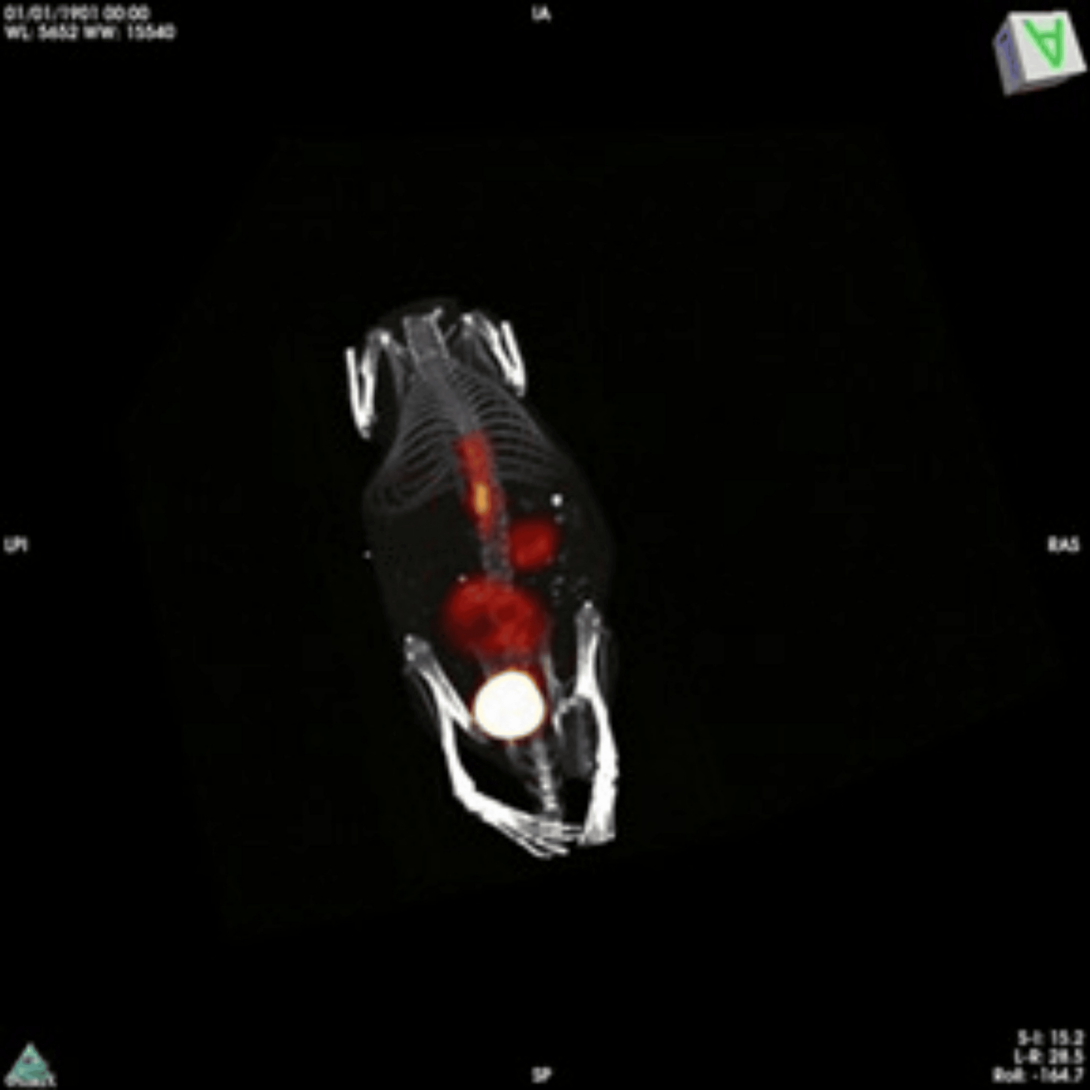
PET-CT performed in the model three weeks post-surgery, showing evidence of 18-FDG uptake as the contrast agent in the lumbar vertebrae at the site where the tumor cells were inoculated, along with areas of external uptake, which are attributed to a probable infectious process.

Subsequently, in the second phase of the study, the mouse-specific cell line 4T1 was used. Eight mice underwent surgery, of which two died, one during the surgical procedure and the other two days after surgery. The remaining six mice developed neurological signs starting in the second week; initially, slight claudication was observed, but without changes in their usual behavior, such as normal food and water intake, no isolation, and no signs of aggression. During the third week, locomotor changes became more evident, with increased claudication and the animal isolating itself in a corner of the cage, as well as a decrease in the intake of solids and liquids. At the beginning of the fourth week, the animal showed difficulty moving, and at that point, the decision was made to terminate it. Most of the animals were sacrificed, except for one, which was used for imaging tests (PET-CT with 18-FDG as a contrast medium) (Figure 3).

**Figure 4:**
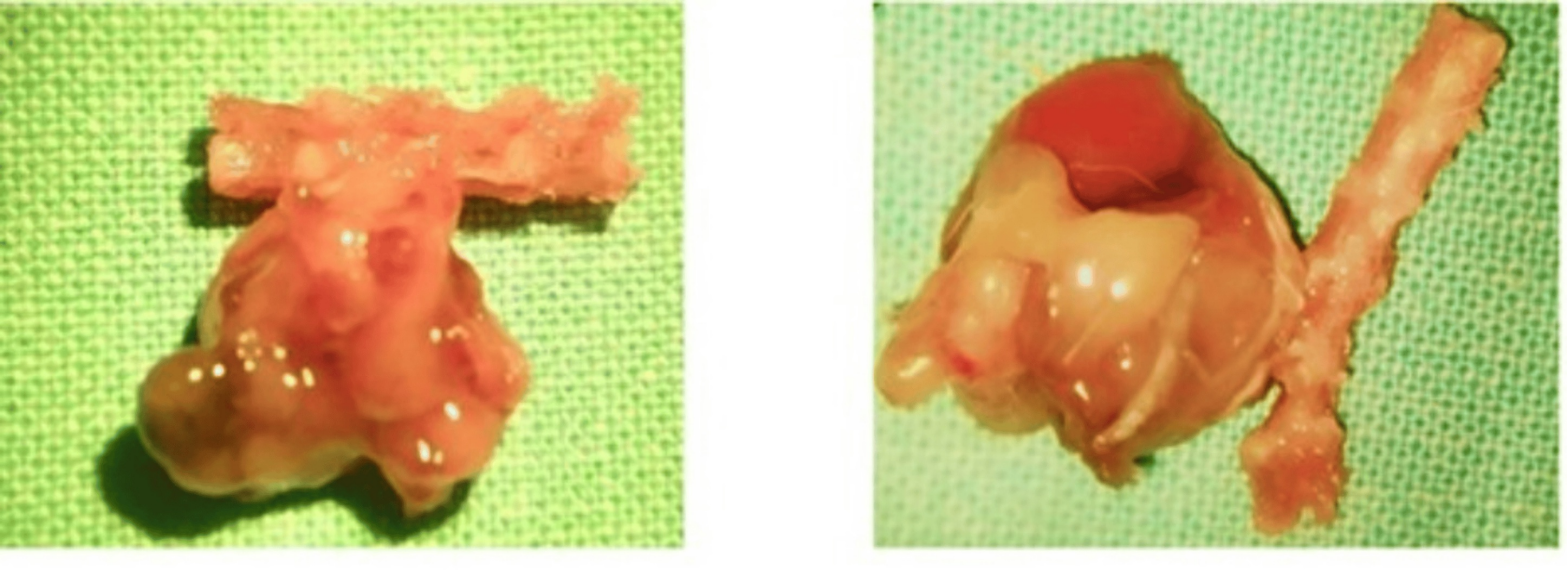
Macroscopic samples obtained after sacrificing the experimental models upon reaching the humanitarian endpoint, showing tumor development in the lumbar vertebrae with invasion into adjacent organs.

**Figure 5:**
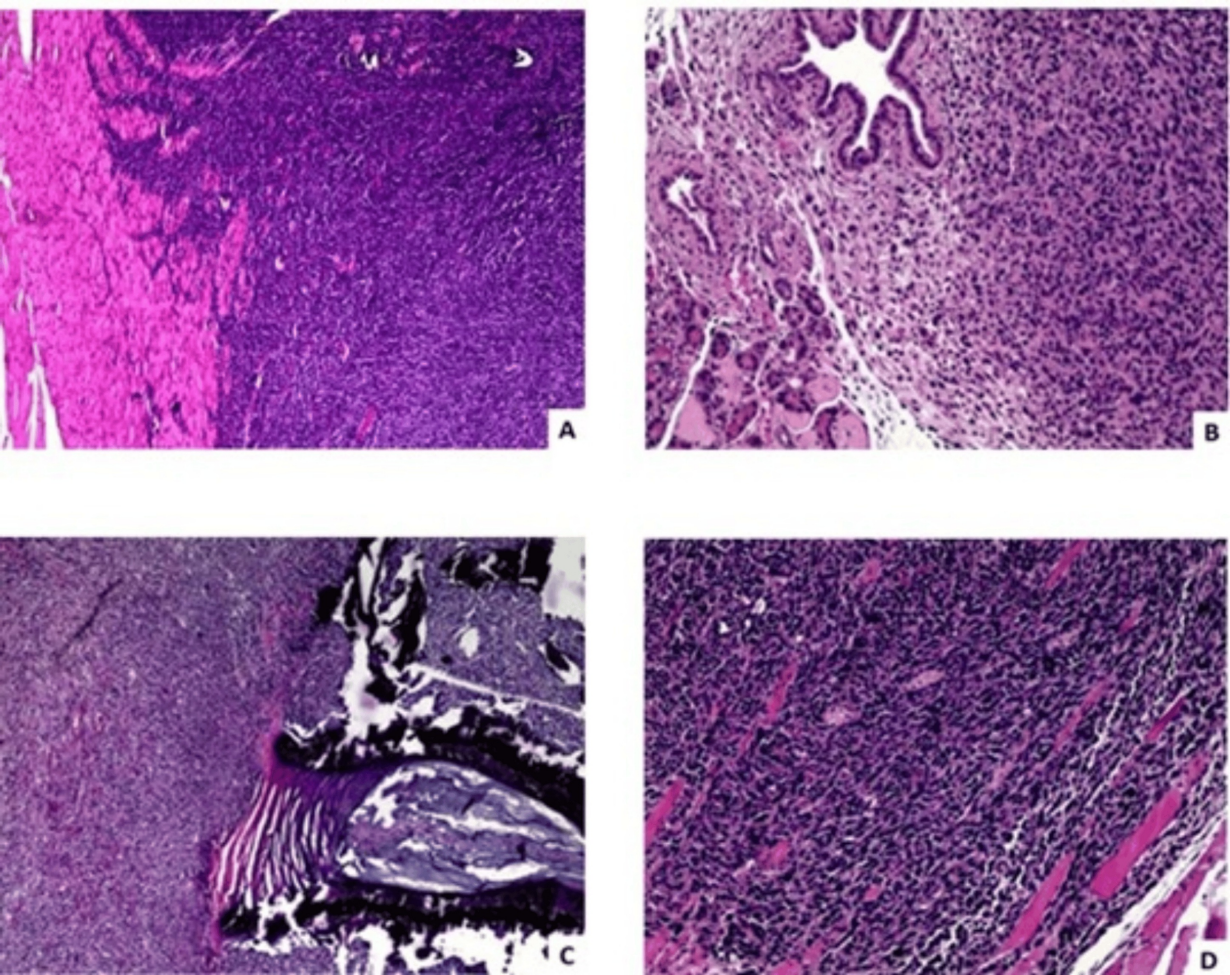
Histopathological sections with H&E staining of the spine of mice inoculated with murine 4T1 breast cancer cells. A) Presence of tumor cells in tissues adjacent to the spine (4x); B) tumor cells in pancreatic tissue, metastatic to the spine (10x); C) tumor cells in the vertebral bodies (4x); D) enlargement of lamella C, showing tumor cells in the striated muscle of the paravertebral musculature (10x).

At necropsy, macroscopic changes were observed (Figure 4) in the lumbar region at the site of tumor cell inoculation, corresponding to the development of masses associated with tumor metastasis. Histopathological studies using H&E staining confirmed the presence of tumor cells (Figure 5).

Once the procedure was performed with favorable results, two additional surgeries were carried out (inoculating only 1,000 cells), during which the experiment was successfully reproduced. The animals exhibited macroscopic and clinical changes (claudication, plantigrade gait, limited mobility) starting in the first week, which worsened by the third week. At this point, the humanitarian endpoint was reached to obtain samples, which confirmed the presence of tumors in the spinal column.

## Discussion

Breast cancer remains a leading cause of cancer-related deaths among women worldwide, and its high propensity for metastasis, particularly to the bone, significantly complicates treatment and prognosis. This study aimed to standardize a murine model of vertebral metastasis using CD1/nu/nu mice, providing valuable insights into the tumor microenvironment and potential therapeutic interventions for spinal metastasis in breast cancer [5,6,14].

The use of the 4T1 murine breast cancer cell line in this study successfully induced vertebral metastasis in CD1/nu/nu mice, demonstrated by PET-CT imaging and histopathological analyses. The choice of 4T1 cells was strategic due to their known aggressive nature and metastatic potential, which closely mimics human breast cancer behavior in murine models [15-17]. Previous studies have similarly employed 4T1 cells to model breast cancer metastasis, but our study specifically focused on spinal involvement, filling a critical gap in current research [18,19].

In contrast, the human MCF-7 cell line did not yield successful metastasis in our model. This aligns with findings by Miller et al. [20], where MCF-7 cells, despite their utility in studying hormone-responsive breast cancer, showed limited metastatic potential in vivo. The differential outcomes underscore the necessity of selecting appropriate cell lines for specific research objectives [21,22]. The successful establishment of metastasis with 4T1 cells supports its continued use in modeling aggressive breast cancer and exploring therapeutic avenues for spinal metastasis [8,12].

Histopathological evaluations revealed distinct differences between the 4T1 and MCF-7 inoculations [16,17,20,23]. The 4T1-inoculated mice exhibited clear evidence of tumor cells within the vertebral bodies, along with adjacent tissue invasion, reflecting aggressive metastatic behavior. These findings are consistent with previous studies where 4T1 cells metastasized to multiple organs, including the lungs and liver, and closely paralleled the pathological features observed in human breast cancer metastasis [5,6,24-26].

The necrotic changes observed in MCF-7-inoculated mice without tumor cell presence align with the cell line's known characteristics. MCF-7 cells often require supplemental factors such as estrogen to maintain growth and metastasis, which might explain their poor performance in inducing metastasis in this study. This aligns with research by Yip et al. [27], who highlighted the need for additional hormonal support for MCF-7 cells in vivo.

Clinical and behavioral observations

Behavioral changes in the mice, particularly those inoculated with 4T1 cells, were significant and indicative of disease progression [27-29]. Early signs of claudication, reduced mobility, and isolation were critical markers of spinal metastasis. These clinical observations mirror those reported by Holen et al. [30], who developed a rodent model of spinal metastasis using human prostate cancer cells. The onset of neurological symptoms and behavioral changes were key indicators of spinal cord compression, underscoring the relevance of such models in studying metastatic spinal cord compression in breast cancer.

Implications for early detection and therapeutic testing

The establishment of this vertebral metastasis model opens avenues for early detection research. Currently, the early diagnosis of bone metastasis remains challenging due to the often asymptomatic nature of the initial stages [31-33]. Molecular markers and imaging advancements, such as those used in this study, could facilitate earlier detection and intervention. Studies have shown the potential of circulating tumor cells (CTCs) and specific biomarkers in predicting metastasis, which could be explored further using this model [34-37].

The model also provides a platform for testing therapeutic strategies aimed at mitigating spinal cord compression and improving patient outcomes [38,39]. The use of PET-CT imaging in this study demonstrated its utility in monitoring disease progression and treatment response. Previous research has validated PET-CT as a reliable tool for assessing metastatic disease in various cancers, including breast cancer [40-42]. The ability to non-invasively track tumor development and response to treatment is crucial for preclinical studies [43-45].

Future directions

Building on this vertebral metastasis model using 4T1 murine breast cancer cells, future work can focus on refining early detection by identifying blood or tissue-based molecular markers for detecting spinal metastasis before overt clinical signs appear [46-49], therapeutic testing by evaluating anti-metastatic agents, targeted therapies, or immunomodulators to prevent or mitigate vertebral involvement [50-53], and gaining mechanistic insights into how tumor cells interact with the bone microenvironment, including examining factors such as cytokine profiles and local immune responses [54-56].

Limitations of the study

While this study successfully established a vertebral metastasis model using the 4T1 murine breast cancer cell line in CD1/nu/nu mice, several limitations must be acknowledged.

Surgical Complications and Mortality Rates

One significant limitation was the high mortality rate observed during the surgical procedures. In the initial phase involving MCF-7 cells, four out of eight mice died, either during surgery or shortly after surgery. Although the mortality rate was reduced in the phase involving 4T1 cells, the loss of animals during surgery highlights the technical challenges and potential variability in surgical skill and execution. These complications can affect the reliability and reproducibility of the model.

Specificity to Certain Cell Lines

This study focused primarily on the 4T1 murine breast cancer cell line, which is known for its aggressive metastatic behavior. While this choice was justified for inducing metastasis, the findings may not be generalizable to other breast cancer cell lines, especially those representing different molecular subtypes. The failure of MCF-7 cells to establish metastasis in this model suggests that the results are specific to highly metastatic cell lines like 4T1 and may not apply to less aggressive or hormone-dependent cancers.

Species-Specific Differences

Murine models, although valuable for preclinical research, have inherent species-specific differences that can limit the direct applicability of findings to human breast cancer. Differences in tumor biology, immune system function, and microenvironmental interactions between mice and humans can affect the translational relevance of the results. While the 4T1 cell line provides a useful model, it may not fully recapitulate the complexity of human breast cancer metastasis.

Limited Scope of Biomarker Analysis

The study primarily focused on establishing the model and validating tumor development through imaging and histopathological analysis. However, it did not delve deeply into the molecular mechanisms or identify specific biomarkers associated with metastasis. A more comprehensive analysis of gene expression, protein markers, and signaling pathways involved in metastatic progression could provide deeper insights and enhance the model's utility for early detection and therapeutic targeting.

Absence of Long-Term Outcome Data

The study's observational period was limited to a few weeks post-inoculation, which may not capture the full progression and long-term impact of vertebral metastasis. Long-term studies are needed to understand the chronic effects of spinal metastasis, including the development of spinal cord compression, neurological deficits, and overall survival. Additionally, the short duration may have prevented the observation of late-stage disease characteristics and treatment responses.

## Conclusions

After histopathology and PET-CT studies, metastasis development was clearly evidenced, along with neurological clinical signs in the model, such as claudication, changes in behavior, water and food intake, isolation, and aggressiveness. We successfully inoculated 1,000 cells from the 4T1 mouse line to develop a bone metastasis model in the spine of breast cancer. Finally, we propose that this model be reproducible in various studies involving clinical, molecular, and prognostic aspects of the evolution of breast cancer spinal metastasis.

## References

[1] Programa de Acción Específico Prevención y Control del Cáncer de la Mujer (2013-2018 (2022). Programa de Acción Específico Prevención y Control del Cáncer de la Mujer (2013-2018). Secretaria de Salud.. Updated 8 de septiembre de.

[2] López CN, Ramón GN, Sánchez MJI, de Santiago GJ (2012). Metástasis óseas múltiples de cáncer de mama: Papel del CA 15.3 y respuesta a la hormonoterapia. Rev Chil Obstet Ginecol.

[3] Zibly Z, Schlaff CD, Gordon I, Munasinghe J, Camphausen KA (2012). A novel rodent model of spinal metastasis and spinal cord compression. BMC Neurosci.

[4] Cacho-Díaz B, García-Botello DR, Wegman-Ostrosky T, Reyes-Soto G, Ortiz-Sánchez E, Herrera-Montalvo LA (2020). Tumor microenvironment differences between primary tumor and brain metastases. J Transl Med.

[5] Kimura T (2018). Multidisciplinary approach for bone metastasis: a review. Cancers (Basel).

[6] Chen YC, Sosnoski DM, Mastro AM (2010). Breast cancer metastasis to the bone: mechanisms of bone loss. Breast Cancer Res.

[7] Holliday DL, Speirs V (2011). Choosing the right cell line for breast cancer research. Breast Cancer Res.

[8] Kondov B, Milenkovikj Z, Kondov G (2018). Presentation of the molecular subtypes of breast cancer detected by immunohistochemistry in surgically treated patients. Open Access Maced J Med Sci.

[9] Jin X, Mu P (2015). Targeting breast cancer metastasis. Breast Cancer (Auckl).

[10] Reyes Soto G, Cacho-Díaza B, Bravo-Reynab C (2023). Prognostic factors associated with overall survival in breast cancer patients with metastatic spinal disease. Cureus.

[11] Pulido C, Vendrell I, Ferreira AR, Casimiro S, Mansinho A, Alho I, Costa L (2017). Bone metastasis risk factors in breast cancer. Ecancermedicalscience.

[12] Yazdani A, Dorri S, Atashi A, Shirafkan H, Zabolinezhad H (2019). Bone metastasis prognostic factors in breast cancer. Breast Cancer (Auckl).

[13] Baklaushev VP, Grinenko NF, Yusubalieva GM (2015). Modeling and integral X-ray, optical, and MRI visualization of multiorgan metastases of orthotopic 4T1 breast carcinoma in BALB/c mice. Bull Exp Biol Med.

[14] Sarabia-Estrada R, Zadnik PL, Molina CA (2013). A rat model of metastatic spinal cord compression using human prostate adenocarcinoma: histopathological and functional analysis. Spine J.

[15] Ng J, Henriquez N, Kitchen N (2024). Suppression of tumour growth from transplanted astrocytoma cells transfected with luciferase in mice by bioluminescence mediated, systemic, photodynamic therapy. Photodiagnosis Photodyn Ther.

[16] Weineisen M, Schottelius M, Simecek J (2015). 68Ga- and 177Lu-labeled PSMA I&T: optimization of a PSMA-targeted theranostic concept and first proof-of-concept human studies. J Nucl Med.

[17] Capulli M, Hristova D, Valbret Z (2019). Notch2 pathway mediates breast cancer cellular dormancy and mobilisation in bone and contributes to haematopoietic stem cell mimicry. Br J Cancer.

[18] Wright LE, Ottewell PD, Rucci N (2016). Murine models of breast cancer bone metastasis. Bonekey Rep.

[19] Liang Y, Zhang H, Song X, Yang Q (2020). Metastatic heterogeneity of breast cancer: Molecular mechanism and potential therapeutic targets. Semin Cancer Biol.

[20] Li X, Liang Y, Lian C (2021). CST6 protein and peptides inhibit breast cancer bone metastasis by suppressing CTSB activity and osteoclastogenesis. Theranostics.

[21] Nolan E, Kang Y, Malanchi I (2023). Mechanisms of organ-specific metastasis of breast cancer. Cold Spring Harb Perspect Med.

[22] Puppo M, Valluru MK, Clézardin P (2021). MicroRNAs and their roles in breast cancer bone metastasis. Curr Osteoporos Rep.

[23] Xu D, Tang M (2023). Advances in the study of biomarkers related to bone metastasis in breast cancer. Br J Radiol.

[24] Yang M, Liu C, Yu X (2019). Skeletal-related adverse events during bone metastasis of breast cancer: current status. Discov Med.

[25] Shin E, Koo JS (2020). The role of adipokines and bone marrow adipocytes in breast cancer bone metastasis. Int J Mol Sci.

[26] Haider MT, Ridlmaier N, Smit DJ, Taipaleenmäki H (2021). Interleukins as mediators of the tumor cell-bone cell crosstalk during the initiation of breast cancer bone metastasis. Int J Mol Sci.

[27] Yip RK, Rimes JS, Capaldo BD (2021). Mammary tumour cells remodel the bone marrow vascular microenvironment to support metastasis. Nat Commun.

[28] Kar S, Katti DR, Katti KS (2020). Bone interface modulates drug resistance in breast cancer bone metastasis. Colloids Surf B Biointerfaces.

[29] Sun J, Hu L, Bok S (2023). A vertebral skeletal stem cell lineage driving metastasis. Nature.

[30] Holen I, Lefley DV, Francis SE, Rennicks S, Bradbury S, Coleman RE, Ottewell P (2016). IL-1 drives breast cancer growth and bone metastasis in vivo. Oncotarget.

[31] Yousefi M, Nosrati R, Salmaninejad A, Dehghani S, Shahryari A, Saberi A (2018). Organ-specific metastasis of breast cancer: molecular and cellular mechanisms underlying lung metastasis. Cell Oncol (Dordr).

[32] Zhou X, Zhu W, Nowicki M (2016). 3D bioprinting a cell-laden bone matrix for breast cancer metastasis study. ACS Appl Mater Interfaces.

[33] Akagi H, Shimada A, Chin K, Domoto H (2021). Successful stabilization of symptomatic bone marrow metastasis two times in a breast cancer patient. Anticancer Res.

[34] Zulauf N, Brüggmann D, Groneberg D, Oremek GM (2019). Expressiveness of bone markers in breast cancer with bone metastases. Oncology.

[35] Batouli A, Braun J, Singh K, Gholamrezanezhad A, Casagranda BU, Alavi A (2018). Diagnosis of non-osseous spinal metastatic disease: the role of PET/CT and PET/MRI. J Neurooncol.

[36] Awosika T, Davidar AD, Hersh AM (2024). SPECT/CT and PET/CT for the evaluation of persistent or recurrent pain after spine surgery: a systematic review and case series. World Neurosurg.

[37] Croset M, Pantano F, Kan CW (2018). miRNA-30 family members inhibit breast cancer invasion, osteomimicry, and bone destruction by directly targeting multiple bone metastasis-associated genes. Cancer Res.

[38] Han W, El Botty R, Montaudon E (2021). In vitro bone metastasis dwelling in a 3D bioengineered niche. Biomaterials.

[39] Johnson RW, Sowder ME, Giaccia AJ (2017). Hypoxia and bone metastatic disease. Curr Osteoporos Rep.

[40] Zhang Z, Wang H, Ikeda S, Fahey F, Bielenberg D, Smits P, Hauschka PV (2010). Notch3 in human breast cancer cell lines regulates osteoblast-cancer cell interactions and osteolytic bone metastasis. Am J Pathol.

[41] Hong S, Youk T, Lee SJ, Kim KM, Vajdic CM (2020). Bone metastasis and skeletal-related events in patients with solid cancer: a Korean nationwide health insurance database study. PLoS One.

[42] Whiteley AE, Ma D, Wang L (2024). Breast cancer exploits neural signaling pathways for bone-to-meninges metastasis. Science.

[43] Beylerli O, Encarnacion Ramirez MJ, Shumadalova A, Ilyasova T, Zemlyanskiy M, Beilerli A, Montemurro N (2023). Cell-free miRNAs as non-invasive biomarkers in brain tumors. Diagnostics (Basel).

[44] Mei S, Alchahin AM, Tsea I (2024). Single-cell analysis of immune and stroma cell remodeling in clear cell renal cell carcinoma primary tumors and bone metastatic lesions. Genome Med.

[45] Reyes Soto G, Cacho-Díaz B, Vilchis-Sámano H (2024). Mexican multicenter experience of metastatic spinal disease. Cureus.

[46] Shin JI, Chee CG, Yoon MA, Chung HW, Lee MH, Lee SH (2024). Vertebral venous congestion that may mimic vertebral metastasis on contrast-enhanced chest computed tomography in Chemoport inserted patients. Korean J Radiol.

[47] Morimoto T, Toda Y, Hakozaki M (2024). A new era in the management of spinal metastasis. Front Oncol.

[48] Yu W, Chen D, Ding X (2024). A critical appraisal of clinical practice guidelines on surgical treatments for spinal metastasis. Eur Spine J.

[49] Takeda K, Umezawa R, Yamamoto T (2024). Lymphopenia after palliative radiotherapy for vertebral metastases. J Radiat Res.

[50] Tsukamoto S, Mavrogenis AF, Masunaga T (2024). Current concepts in the treatment of giant cell tumor of bone: an update. Curr Oncol.

[51] Gareev I, Encarnacion Ramirez MJ, Nurmukhametov R (2023). The role and clinical relevance of long non-coding RNAs in glioma. Noncoding RNA Res.

[52] Piffko A, Uhl C, Vajkoczy P, Czabanka M, Broggini T (2022). ephrinB2-EphB4 signaling in neurooncological disease. Int J Mol Sci.

[53] Wu MY, Li CJ, Yiang GT (2018). Molecular regulation of bone metastasis pathogenesis. Cell Physiol Biochem.

[54] Gareev I, de Jesus Encarnacion Ramirez M, Goncharov E, Ivliev D, Shumadalova A, Ilyasova T, Wang C (2023). MiRNAs and lncRNAs in the regulation of innate immune signaling. Noncoding RNA Res.

[55] Lo VC, Akens MK, Moore S, Yee AJ, Wilson BC, Whyne CM (2012). Beyond radiation therapy: photodynamic therapy maintains structural integrity of irradiated healthy and metastatically involved vertebrae in a pre-clinical in vivo model. Breast Cancer Res Treat.

[56] Bergin S, Than KD (2022). Advances in the treatment of spinal metastasis: commentary on “spinal metastases and the evolving role of molecular targeted therapy, chemotherapy, and immunotherapy”. Neurospine.

